# Response of the Biocontrol Agent Pseudomonas pseudoalcaligenes AVO110 to Rosellinia necatrix Exudate

**DOI:** 10.1128/AEM.01741-18

**Published:** 2019-01-23

**Authors:** Clara Pliego, José Ignacio Crespo-Gómez, Adrián Pintado, Isabel Pérez-Martínez, Antonio de Vicente, Francisco M. Cazorla, Cayo Ramos

**Affiliations:** a Área de Genética, Facultad de Ciencias, Universidad de Málaga, Málaga, Spain; b Instituto de Hortofruticultura Subtropical y Mediterránea La Mayora, Consejo Superior de Investigaciones Científicas (IHSM-UMA-CSIC), Málaga, Spain; c IFAPA-Centro de Málaga, Plant Breeding and Biotechnology Department, Málaga, Spain; d Departamento de Microbiología, Facultad de Ciencias, Universidad de Málaga, Málaga, Spain; Technische Universität Wien

**Keywords:** avocado plants, *Pseudomonas pseudoalcaligenes*, *Rosellinia necatrix*, biocontrol, fungal exudate, mycelium colonization, rhizosphere colonization, signature-tagged mutagenesis

## Abstract

The rhizobacterium Pseudomonas pseudoalcaligenes AVO110, isolated by the enrichment of competitive avocado root tip colonizers, controls avocado white root rot disease caused by Rosellinia necatrix. Here, we applied signature-tagged mutagenesis (STM) during the growth and survival of AVO110 in fungal exudate-containing medium with the goal of identifying the molecular mechanisms linked to the interaction of this bacterium with R. necatrix. A total of 26 STM mutants outcompeted by the parental strain in fungal exudate, but not in rich medium, were selected and named growth-attenuated mutants (GAMs). Twenty-one genes were identified as being required for this bacterial-fungal interaction, including membrane transporters, transcriptional regulators, and genes related to the metabolism of hydrocarbons, amino acids, fatty acids, and aromatic compounds. The bacterial traits identified here that are involved in the colonization of fungal hyphae include proteins involved in membrane maintenance (a dynamin-like protein and ColS) or cyclic-di-GMP signaling and chemotaxis. In addition, genes encoding a DNA helicase (*recB*) and a regulator of alginate production (*algQ*) were identified as being required for efficient colonization of the avocado rhizosphere.

**IMPORTANCE** Diseases associated with fungal root invasion cause a significant loss of fruit tree production worldwide. The bacterium Pseudomonas pseudoalcaligenes AVO110 controls avocado white root rot disease caused by Rosellinia necatrix by using mechanisms involving competition for nutrients and niches. Here, a functional genomics approach was conducted to identify the bacterial traits involved in the interaction with this fungal pathogen. Our results contribute to a better understanding of the multitrophic interactions established among bacterial biocontrol agents, the plant rhizosphere, and the mycelia of soilborne pathogens.

## Abstract (precis)

Diseases associated with fungal root invasion cause a significant loss of fruit tree production worldwide. The bacterium Pseudomonas pseudoalcaligenes AVO110 controls avocado white root rot disease caused by Rosellinia necatrix by using mechanisms involving competition for nutrients and niches. Here, a functional genomics approach was conducted to identify the bacterial traits involved in the interaction with this fungal pathogen. Our results contribute to a better understanding of the multitrophic interactions established among bacterial biocontrol agents, the plant rhizosphere, and the mycelia of soilborne pathogens.

## INTRODUCTION

The significance of multitrophic interactions established among organisms during the biological control of soilborne plant pathogens has been extensively reported (1–3), and it is widely accepted that successful colonization of the plant rhizosphere is a relevant biocontrol trait (4). In this regard, many studies have focused on the identification of bacterial genes involved in the attachment and colonization of plant roots and seeds, with special emphasis on biocontrol *Pseudomonas* (5–9). However, little attention has been given to the trophic and physical interactions established between bacterial biocontrol agents and root pathogenic fungi. Biocontrol *Pseudomonas* spp. have been shown to interact with fungal pathogens via chemotaxis and by living in close proximity to fungi, often colonizing hyphal surfaces and utilizing nutrients exuded from living fungal cells (1, 10). In fact, this bacterial behavior has been proposed to contribute to biocontrol through the biosynthesis of antifungal compounds or the release of enzymes involved in the degradation or alteration of fungal components (11).

The biocontrol rhizobacterium Pseudomonas pseudoalcaligenes (*Proteobacteria*, *Gammaproteobacteria*, *Pseudomonadales*) strain AVO110, isolated by the enrichment of competitive avocado (Persea americana) root tip colonizers, is able to control avocado white root rot disease caused by the soilborne pathogen Rosellinia necatrix (Ascomycota, Sordariomycetes, Xylariales) under greenhouse conditions, reducing disease development up to 45% when compared to that in control plants not inoculated with bacteria (12). Although most bacterial strains isolated by using this strategy were shown to produce several exoenzymes, hydrogen cyanide (HCN), or antifungal antibiotics, the potential biocontrol traits of P. pseudoalcaligenes AVO110 only included the biosynthesis of siderophores, weak cellulose activity (12), and its ability to colonize both the avocado rhizosphere and the R. necatrix hyphae (1). Thus, competition for nutrients and niches was proposed as the most relevant biocontrol trait of this bacterium (1). Actively growing fungal hyphae exude a complex mixture of low-molecular-weight metabolites that include organic acids, such as oxalic, citric, and acetic acids, peptides, amino acids, sugars, and sugar alcohols such as mannitol (13–16). Along this line, P. pseudoalcaligenes AVO110 was shown to efficiently grow on minimal BM medium only when supplemented with R. necatrix exudates (BM-RE medium), reaching higher cell densities than other nonbiocontrol rhizobacterial strains (1). Thus, AVO110 might harbor specific traits conferring a competitive advantage to this bacterium during its interaction with fungi.

Several strategies are currently available to unravel the bacterial genes involved in host interactions, including transcriptional profiling, *in vivo* expression technology (IVET), and signature-tagged mutagenesis (STM) (17–19). However, few studies have addressed the identification of bacterial genes involved in interactions with fungal phytopathogens. During a commensal interaction, the plant-pathogenic fungus Gaeumannomyces graminis was shown to induce Pseudomonas fluorescens Pf29Arp genes involved in mycelium colonization even before cell-to-cell contact (20). Likewise, in a noncontact confrontation assay, the mycophagous bacterium Collimonas fungivorans responded to Aspergillus niger by activating genes for the utilization of fungal derived compounds and for production of a putative antifungal compound (21). In two studies that applied IVET, Pseudomonas putida strain 06909 genes induced during the colonization of *Phytophthora* mycelia were identified (22, 23). This strategy yielded several genes involved in carbon and amino acid metabolism, ABC transporters, and outer membrane porins. Furthermore, the colonization of A. niger hyphae by Bacillus subtilis was suggested to be an active process in which the bacterium rewires not only surface attachment, but also metabolism, motility, general stress responses, and antimicrobial production (24). Despite all these exciting new insights into the interactions established between bacteria and fungi, the genes revealed by transcriptional profiling or IVET should be further investigated experimentally by functional approaches. In this sense, STM combines the power of insertional mutagenesis and negative selection with a detection system, which allows the identification of individual attenuated mutants from a complex mutant pool. Previously, we showed that the utilization of fungal exudates plays an important role in the biocontrol ability of P. pseudoalcaligenes AVO110 against R. necatrix (1). In this study, we used this bacterial-fungal model system to apply STM in the identification of bacterial genes involved in interactions with a fungal phytopathogen. Sequence analysis of the genes interrupted by the transposon in the selected P. pseudoalcaligenes AVO110 mutants revealed several molecular processes involved in the interactions of this bacterium with R. necatrix, including genes related to the colonization of biological surfaces and the utilization of fungal exudates.

## RESULTS

### Selection of Pseudomonas pseudoalcaligenes AVO110 growth-attenuated mutants in Rosellinia necatrix exudates.

A mutant bank of 3,408 P. pseudoalcaligenes AVO110 mini-Tn*5*Km2-tagged derivatives was constructed as described in Materials and Methods using the strains listed in Table 1. Grouped in 76 input pools of ≤45 mutants, all 3,408 STM mutants were screened for growth in minimal BM medium containing R. necatrix exudates (BM-RE medium, pH 7.11). All input pools included a negative control (wild-type P. pseudoalcaligenes AVO110) and a positive control (a mini-Tn*5*Km2-tagged AVO110 derivative selected by its ability to grow and survive on both LB and BM-RE medium). A total of 765 strains showing stronger hybridization signals with the input probe than with the output probe were selected for further characterization. To reduce the number of false-positive candidates, the selected mutants were grouped in new pools, mixed with other random mutants, and retested in a second round of STM screening. After this second STM round, the number of mutants was reduced to 99 strains (see Fig. S1 in the supplemental material).

**TABLE 1:** Strains and plasmids used in this study

Strain or plasmid	Relevant characteristics	Reference or source
Strains		
Bacteria^*a*^		
Pseudomonas pseudoalcaligenes		
AVO110	Wild-type strain	12
AVO110-Km	AVO110 tagged with mini-Tn*7*-km, also carrying *gfp*	1
GAM2-Gm	GAM2 (Km^r^) tagged with mini-Tn*7*Gm, also carrying *gfp*	This study
GAM3-Gm	GAM3 (Km^r^) tagged with mini-Tn*7*Gm, also carrying *gfp*	This study
GAM22-Gm	GAM22 (Km^r^) tagged with mini-Tn*7*Gm, also carrying *gfp*	This study
GAM24-Gm	GAM24 (Km^r^) tagged with mini-Tn*7*Gm, also carrying *gfp*	This study
GAM26-Gm	GAM26 (Km^r^) tagged with mini-Tn*7*Gm, also carrying *gfp*	This study
Escherichia coli		
XL1-Blue	*hsdR17 supE44 recA1 endA1 gyrA46 thi relA1 lac* [F′ *proAB*^+^ *lacI*^q^, *lacZ*ΔM15::Tn*10*(Tc^r^)]	83
DH5α	F^−^ φ80d*lac*ZΔM15 Δ(*lacZYA-argF*)*U169 deoR recA1 endA1 hsdR17*(r_K_^−^ m_K_^+^) *phoA supE44* λ^−^ *thi-1 g*y*rA96 relA1*	70
S17-1 λ*pir*	*thi pro hsdR recA* RP4-2 (Tc::Mu Km::Tn*7*)λTc^r^ Str^r^, *pir* lysogen	84
CC118(λ*pir*)	Δ(*ara-leu*) *araD* Δ*lacX74 galE galK phoA20 thi-1 rpsE rpoB argE*(Am) *recA1*, λ*pir* lysogen (Rif^r^)	85
HB101	Sm^r^ *recA thi pro leu hsdRM^+^*, used for replication of the helper plasmid for RK600	86
SM10::λ*pir*	*thi-1 thr leu tonA lacY supE recA*::RP4-2-Tc::Mu, Km^r^, λ*pir*, used for replication of pUX-BF13	79
Fungi		
Rosellinia necatrix Rn400	Wild type, high virulence on avocado	87
Plasmids		
pUTmini-Tn*5*Km2-STM	Pool of tagged pUTmini-Tn*5*Km2 vectors (Amp^r^, Km^r^)	17
pBluescript II SK(-)	Cloning vector; orif1(−), oripUC, P*_lac_*, *lacZ*′ (Amp^r^)	Agilent Technologies, Inc., Santa Clara, CA, USA
pGEM-T Easy vector	PCR cloning vector, 3′-T ends (Amp^r^)	Promega Corp., Madison, WI, USA
pRK600	Cm^r^ ori-ColE1 RJ2-mob^+^ RJ2^−^ TRA^+^ helper plasmid in matings	86
pUX-BF13	Amp^r^ mob^+^ ori-R6K helper plasmid providing the Tn*7* transposition functions in *trans*	79
pBK-mini-Tn*7*(Gm)-P_A1/04/03_-gfp2	Puc19-based delivery plasmid for mini-Tn*7-gfp2*, Gm^r^, Amp^r^, Cm^r^, Sm^r^, Mob*, P_A1/04/03_, constitutive P*_lac_* derivative promoter	80

a Strains GAM2, GAM3, GAM22, GAM24, and GAM26, which are *P. pseudoalcaligenes* AVO110 derivatives containing a mini-Tn*5*Km2 transposon carrying a kanamycin resistance (Km^r^) gene, are described in Table 2.

**FIG 1:**
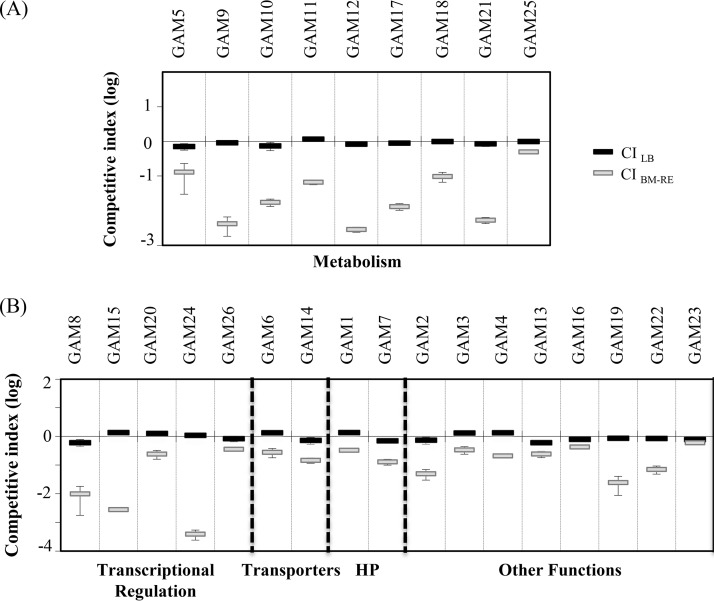
Competition assays of Pseudomonas pseudoalcaligenes growth-attenuated mutant (GAM) strains. Competitive index values (CI) are shown for mixed inoculations of P. pseudoalcaligenes and its derivative GAM strain. CI_LB_, competition index values in lysogenic broth (LB) medium. CI_BM-RE_, competition index values in minimal BM medium supplemented with R. necatrix exudates (BM-RE medium). CI assays of GAM strains disrupted in metabolism-related (A) and non-metabolism-related (B) genes. CIs are the means from three samples, and the error bars represent the standard deviations from the averages. In all cases, CI_BM-RE_ was significantly less than 1.0 and significantly lower than CI_LB_. Statistical analyses were performed by Student's *t* tests.

Single insertion of the transposon into the genome of each mutant was determined by the hybridization of EcoRI-BglII-digested total DNA against a transposon probe (*aphA* gene). Of the 99 mutant strains, 85 contained a single insertion of the transposon and were selected for further analysis (see Materials and Methods). These strains were potentially mutated either in essential genes or in genomic regions required for competitive survival in BM-RE medium. To differentiate between these two possibilities, competitive indexes (CIs) of each of the selected mutants in comparison with the wild-type strain were calculated both in LB medium (CI_LB_) and in BM-RE medium (CI_BM-RE_). A total of 26 mutants showed CI_BM-RE_ and CI_LB_ values significantly less than 1 and not significantly different from 1, respectively (CI_BM-RE_ < 1 and CI_LB_ = 1) (Fig. 1). Thus, these strains, which were named growth-attenuated mutants (GAMs), were outcompeted by the wild-type strain only in medium supplemented with fungal exudates.

### Pseudomonas pseudoalcaligenes AVO110 genes required for growth in fungal exudates.

Genomic DNA fragments flanking the transposon insertion within the 26 selected GAM strains were cloned, sequenced, and used to search the RAST-annotated draft genome sequence of P. pseudoalcaligenes AVO110 generated in this study (see Materials and Methods). Furthermore, the nucleotide sequences of the interrupted AVO110 genomic regions were used to search the GenBank and ASAP databases. After the identification of several independent insertions of the transposon in the same gene (*colS*, *leuC*, and *recB*) and insertions in intergenic regions (strains GAM13 and GAM19), a total of 21 different genes were identified and classified into five categories according to the putative gene function of the highest-quality BLASTp alignment (Table 2).

**TABLE 2:**
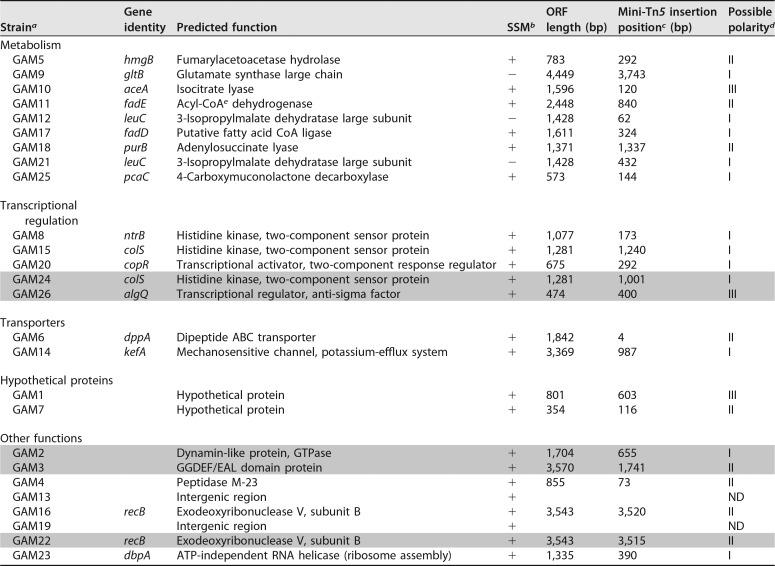
*Pseudomonas pseudoalcaligenes* AVO110 GAMs selected by STM

^a^Shading indicates growth-attenuated mutant (GAM) strains selected for further characterization according to the predicted function of the gene interrupted by the transposon.

^b^Ability (+) or inability (−) to grow in standard succinate medium (SSM) minimal medium.

^c^Mini-Tn*5*Km2 insertion point. Numbers indicate the exact position of the transposon in the disrupted gene, assuming the first nucleotide of the start codon is 1.

^d^Possible polarity of mini-Tn*5*Km2 insertions in growth-attenuated mutant (GAM) strains. Operon predictions were performed according to the criteria defined in reference 30. I, intergenic distances of <40 nucleotides (nt) between the genes interrupted by the transposons and the next downstream genes were considered to affect operonic pairs; II, distances >40 but <200 nt were considered to affect operonic pairs with intermediate probability; III, at distances >200 nt, mutations were considered to affect probable single loci (genes interrupted by the transposon located upstream of a gene transcribed in the opposite orientation were also included in this category); ND, not determined (the transposon was located within an intergenic region).

^e^CoA, coenzyme A.

GAM strains affected in metabolism-related genes (9 strains, 35.6% of the total) showed CI_BM-RE_ values ranging from approximately 10^−3^ to 10^−1^ (Fig. 1). Four of these mutants showed disruptions in genes related to the biosynthetic pathways of purine (GAM18, *purB*) and the amino acids glutamate (GAM9, *gltB*) and leucine (GAM12 and GAM21, *leuC*). Strains GAM9, GAM12, and GAM21 were unable to grow on minimal standard succinate medium (SSM) plates (Table 2) but grew on rich LB medium (Fig. 1) and were therefore considered auxotrophs. Our screening also identified genes encoding enzymes involved in the β-oxidation of fatty acids (*fadE* and *fadD*), the degradation of the aromatic amino acids phenylalanine and tyrosine (*hmgB*), the catabolism of hydroxybenzoate (*pcaC*), and the assimilation of acetate, acyclic terpenes, and leucine (*aceA*) (Table 2).

Transposon insertions in 5 of the 26 selected mutants were located in putative regulatory genes (CI_BM-RE_ values from >10^−4^ to approximately 10^−1^). The interrupted genes in these strains are related to the regulation of the response to changes in nitrogen balance (*ntrB*) (25), the regulation of membrane functionality (*colS*) (26), metal resistance (*copR*) (27), and the regulation of both alginate production and quorum sensing (*algQ*) (28, 29). Higher CI_BM-RE_ values (>10^−2^) were obtained for strains GAM6 (*dppA*) and GAM14 (*kefA*), which were affected in genes encoding components of putative transporters, i.e., a dipeptide ABC transporter and a mechanosensitive channel involved in potassium efflux, respectively. The other genes interrupted by the transposon in GAM strains encode hypothetical proteins, nucleic acid-related proteins, such as RecB and DbpA, a putative dynamin-like protein (DLP), a GGDEF/EAL domain-containing protein, and a peptidase M23 family protein (Table 2).

### DNA context analysis of transposon insertions in the genome of Pseudomonas pseudoalcaligenes AVO110.

The genetic context surrounding each of the sequences interrupted by the transposon in GAM strains was analyzed. Special attention was given to the possible polar effect of the transposon insertions in the transcription of downstream genes (30). Of the 24 open reading frames (ORFs) interrupted by the transposons, 9 were considered to possibly form operons (Table 2).

Our STM approach identified several genes previously highlighted for their relevant role in rhizosphere colonization and competitiveness, such as *recB* (GAM22), *colS* (GAM24), and *algQ* (GAM26) (31, 32). Taking into account that the biocontrol ability of P. pseudoalcaligenes AVO110 has been related to its ability to efficiently colonize avocado roots and R. necatrix hyphae, these three mutants were selected for further genetic characterization. Strains GAM2 and GAM3, which contain the transposon in genes encoding a putative DLP and a GGDEF/EAL domain-containing protein, respectively, were also selected for these analyses. These two genes especially caught our attention because of their possible role in bacterial-host interactions. Bacterial DLPs have been suggested to play a role in membrane remodeling under environmental stresses (33, 34). On the other hand, GGDEF/EAL domain-containing proteins were previously found to be related to rhizosphere colonization in Pseudomonas putida (35) and P. fluorescens (36).

**FIG 2:**
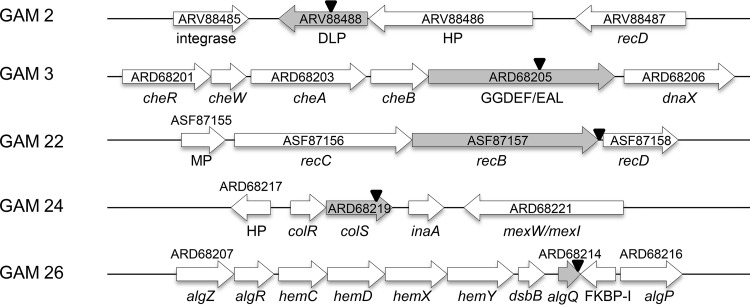
DNA context of transposon insertions in Pseudomonas pseudoalcaligenes AVO110 growth-attenuated mutants (GAMs). Arrows indicate the direction of transcription and relative sizes of the genes in the genome of P. pseudoalcaligenes AVO110. Numbers within or over the arrows designate GenBank accession numbers. Gray arrows indicate the gene interrupted by the mini-Tn*5* transposon. The inverted black triangles indicate the position where the transposon was integrated in the corresponding GAM mutant. Genes whose closest homologs are currently unnamed are indicated by the possible function of their corresponding encoded proteins as follows: DLP, dynamin-like protein; HP, hypothetical protein; GGDEF/EAL, GGDEF/EAL domain protein; MP, membrane protein; FKBP-I, FKBP-type peptidyl-prolyl *cis-trans* isomerase.

Figure 2 summarizes the genetic context of the genes interrupted by the transposon in the five selected GAM strains. RAST annotation identified the protein encoded by the gene interrupted in GAM2 as an ortholog of Escherichia coli LeoA, a GTPase domain-containing DLP (37). This DLP-encoding gene was found to overlap 1 bp with its upstream gene encoding a hypothetical protein. In accordance with the localization of the *leoABC* operon in an E. coli strain H10407 pathogenicity island (38), a gene encoding a phage integrase was detected downstream this putative operon in AVO110 (Fig. 2).

The gene interrupted by the transposon in GAM3 possibly forms an operon with four ORFs showing homology (coverage, 95% to 100%; identity, 59% to 77%) with several *Pseudomonas* species genes involved in flagellar motility/chemotaxis (*cheR*, *cheW*, *cheA*, and *cheB*). On the other hand, and in agreement with the operon organization of *recB*, *recC*, and *recD* in other bacteria (39), the AVO110 *recB* gene, interrupted in GAM22, was also found in the proximity of *recC* and *recD*. This was also the case for the *colS* gene, interrupted in GAM24, which encodes the sensor protein of the two-component system ColR/ColS. Although the insertion of the transposon in GAM26 possibly affects a single locus (*algQ*), other genes related to the production of exopolysaccharides (*algZ*, *algR*, and *algP*) and porphyrins (*hemC*, *hemD*, *hemX*, and *hemY*) were found in the proximity of this gene (Table 2, Fig. 2).

### Modulation of transcript levels in wild-type Pseudomonas pseudoalcaligenes AVO110 after transfer to fungal exudate-containing medium.

**FIG 3:**
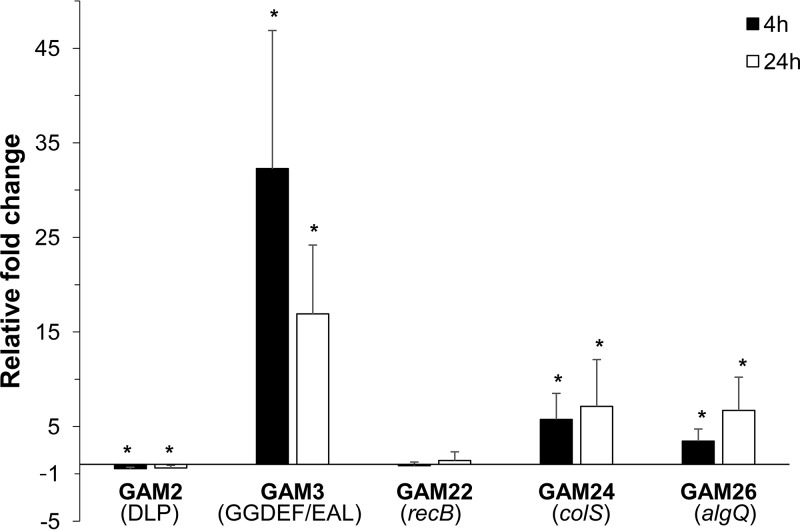
Expression of five selected genes in wild-type Pseudomonas pseudoalcaligenes AVO110 after transfer to *Rosellinia* exudate-containing medium (BM-RE medium). The expression of the indicated genes (DLP [dynamin-like protein gene], GGDEF/EAL [GGDEF/EAL domain-encoding gene], *recB*, *colS*, and *algQ*) was measured by qRT-PCR in AVO110 at 4 h and 24 h after transfer to BM-RE medium. The fold change was calculated after normalization using the housekeeping *rpoD* gene as an internal control. After the normalization, expression fold changes at 4 h and 24 h were calculated with respect to gene expression obtained before the transfer to BM-RE medium (time zero). qRT-PCR values are the means from three biological replicates with three technical replicates. Bars represent the standard deviations from the averages. Statistical analyses were performed by Student’s *t* tests. *, value deviates significantly (*P* < 0.05) from unity (i.e., significantly different from the time zero value).

Expression of the genes interrupted by the transposon in the five selected GAM strains (GAM2, GAM3, GAM22, GAM24, and GAM26) was analyzed after transfer of wild-type P. pseudoalcaligenes AVO110 cells to fungal exudate-containing (BM-RE) medium. For this purpose, AVO110 cells were grown in LB medium to an optical density at 600 nm (OD_600_) of 0.5, washed, and then transferred to BM-RE medium. Samples for RNA extraction were taken immediately after the transfer to BM-RE medium (time zero) and after 4 h and 24 h of incubation. Figure 3 shows the expression of these five genes, normalized to the housekeeping gene *rpoD*, in BM-RE medium relative to their expression at time zero (relative fold changes). While the levels of *recB* transcripts did not change after the transfer of AVO110 cells to BM-RE medium, the transcript levels of the DLP-encoding gene were reduced by half at both 4 h and 24 h after transfer to BM-RE medium. However, the transcript levels of the remaining three genes increased after transfer to the fungal exudate-containing medium. The sharpest increase in transcript levels was observed for the GGDEF/EAL domain-encoding gene, which showed values that were approximately 32 and 17 times higher at 4 h and 24 h, respectively, after transfer to the BM-RE medium (Fig. 3).

### Altered colonization of R. necatrix mycelia by Pseudomonas pseudoalcaligenes GAM strains.

**FIG 4:**
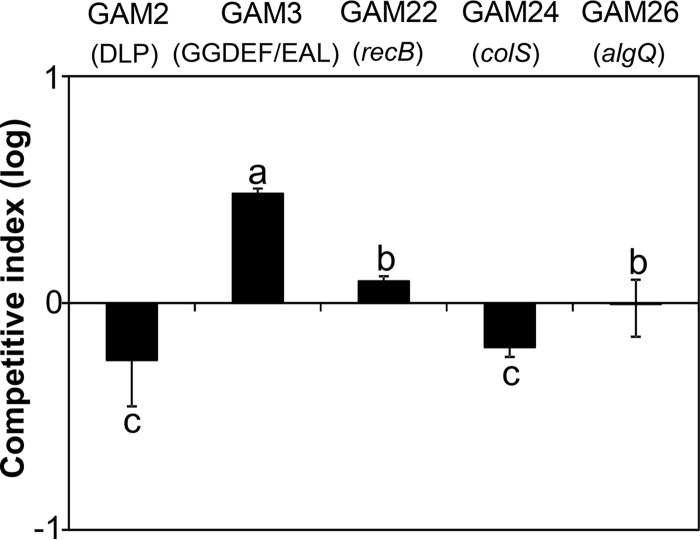
Competition assays between Pseudomonas pseudoalcaligenes AVO110 growth-attenuated mutants (GAM strains) and the wild-type strain during colonization of R. necatrix mycelia. Competitive index (CI) values are shown for mixed inoculations of P. pseudoalcaligenes AVO110 and its derivative GAM strain in minimal medium BM plates covered by R. necatrix mycelia. The CIs shown are the means from three samples, and the error bars represent the standard errors. Different lowercase letters denote significant differences (*P* < 0.05) using one-way ANOVA followed by Tukey’s HSD test with the correction of Bonferroni.

The competitiveness of the selected P. pseudoalcaligenes GAM strains after inoculating over R. necatrix mycelia was tested in competition assays with the wild-type strain. GAM strains (Nf^r^ Km^r^) were differentiated from P. pseudoalcaligenes AVO110 (Nf^r^) using plates containing kanamycin (Km). Mixed inocula of AVO110 and each of the five GAM mutants were prepared and used to inoculate R. necatrix mycelia grown on BM plates. Bacteria were recovered from the fungal hyphae 6 days after incubating at 25°C. After this period, the total number of P. pseudoalcaligenes cells recovered from the fungal mycelia ranged in all cases between 10^4^ to 10^5^ CFU · g^−1^ of mycelia. Considering that P. pseudoalcaligenes AVO110 is unable to grow in this medium in the absence of R. necatrix mycelia or fungal exudates (1), the number of CFU recovered for each of the strains reflects their growth and survival under the influence of the fungal mycelia. While strain GAM3 (GGDEF/EAL mutant) was more competitive than AVO110 (CI > 1) during colonization of fungal hyphae, strains GAM2 and GAM24, which were affected in the DLP-encoding gene and the *colS* gene, respectively, were outcompeted by the wild-type strain (CI > 1). Finally, strains GAM22 (*recB*) and GAM26 (*algQ*) showed CI values that were not significantly different from 1, indicating that they were as competitive as AVO110 (Fig. 4).

### Altered colonization of avocado roots by Pseudomonas pseudoalcaligenes GAM strains.

Wild-type P. pseudoalcaligenes AVO110 was tagged with a mini-Tn*7* derivative (mini-Tn*7*Km) carrying a Km resistance gene and the *gfp* gene (AVO110-Km) (Table 1). P. pseudoalcaligenes GAM strains, which were already resistant to Km due to the insertion of the mini-Tn*5*Km2 derivative, were tagged with mini-Tn*7*Gm, which carries a gentamicin (Gm) resistance gene and the *gfp* gene (Table 1). Competition assays between AVO110-Km and each of the Gm-tagged GAM mutants (GAM-Gm) during growth on LB medium revealed that all GAM-Gm strains were as competitive as AVO110-Km, suggesting that the expression of double antibiotic resistance by GAM-Gm strains does not affect bacterial fitness under these conditions (see Fig. S2).

**FIG 5:**
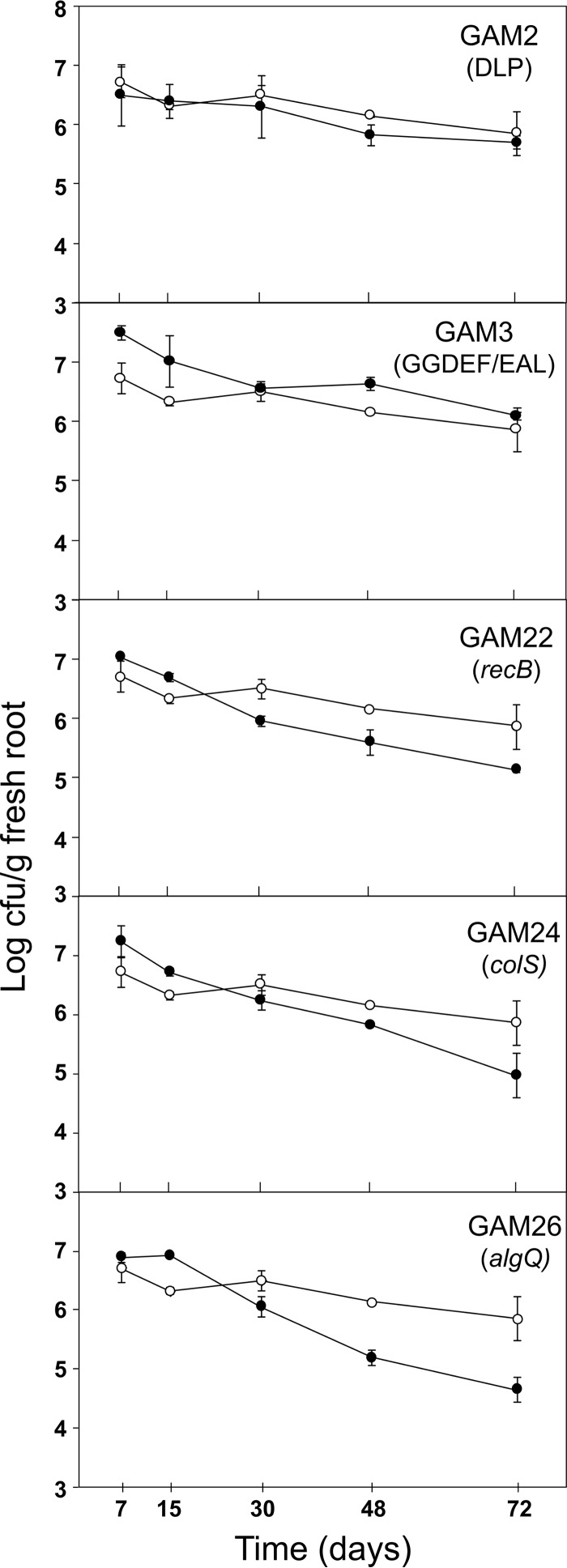
Colonization of avocado roots by Pseudomonas pseudoalcaligenes growth-attenuated mutants (GAMs). Avocado seedlings (cv. Walter Hole) were inoculated with bacterial suspensions (10^3^ to 10^4^ CFU · ml^−1^) of P. pseudoalcaligenes AVO110-kanamycin (Km) or its GAM-gentamicin (Gm) derivative strains (Table 1). Bacteria were recovered from the roots at 7, 15, 30, 48, and 72 days after inoculation and plated on LB agar supplemented with nitrofurantoin (Nf) and Gm for counts of GAM-Gm strains (•) and Nf and Km for counts of the wild-type derivative strain AVO110-Km (○). Data represent the averages from at least three independent plants per sampling point ± standard errors.

P. pseudoalcaligenes AVO110-Km and each of the constructed GAM-Gm strains (Table 1) were individually inoculated in the roots of commercial 6-month-old avocado seedlings (P. americana cv. Walter Hole). At 7, 15, 30, 48, and 72 days after inoculating, the bacteria were extracted from the avocado roots and plated on LB-Km (AVO110-Km) or LB-Km-Gm (GAM-Gm strains). Codification of the *gfp* gene within the mini-Tn*7* derivatives used to construct these strains facilitated the tracking of P. pseudoalcaligenes colonies and their differentiation from other rhizosphere bacteria. All strains were able to establish in the root system of avocado seedlings during the first week postinoculation, reaching approximately 10^7^ CFU · g^−1^ fresh root at 7 days postinoculation (dpi). Thereafter, bacterial counts for AVO110-Km slowly decreased to approximately 10^6^ CFU · g^−1^ fresh root at the end of the experiment (72 dpi). These results are in agreement with the previously reported persistence of wild-type AVO110 on the roots of avocado plants (12), indicating that root colonization by AVO110-Km is not affected by the mini-Tn*7*Km transposon. A similar ability to establish and survive in the avocado rhizosphere was observed for strain GAM2-Gm (DLP mutant). In contrast, a faster decline in CFU counts was observed for strains GAM22-Gm (*recB* mutant), GAM24-Gm (*colS* mutant), and GAM26-Gm (*algQ* mutant), which showed 10^4^ to 10^5^ CFU · g^−1^ fresh root at 72 dpi. On the other hand, strain GAM3-Gm (GGDEF/EAL mutant) established in the avocado rhizosphere at slightly higher cell counts than AVO110-Km at almost all sampling times. However, both strains reached similar cell densities at the end of the experiment (Fig. 5). Together, these results revealed the differences between the colonization ability of the wild-type derivative AVO110-Km and those of some of the analyzed GAM-Gm strains. While strain GAM3-Gm (GGDEF/EAL mutant) showed a slightly higher ability than AVO110-Km to persist in the avocado rhizosphere, GAM strains affected in *recB*, *colS*, and *algQ* showed a lower colonization ability than the wild-type derivative.

### The GGDEF/EAL domain-encoding gene interrupted in GAM3 forms a transcriptional unit with a *cheRWAB* gene cluster.

**FIG 6:**
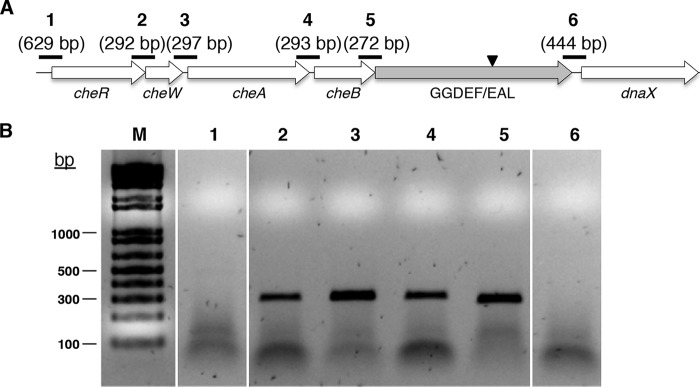
Transcriptional analysis of the *cheRWAB*-GGDEF/EAL operon in Pseudomonas pseudoalcaligenes AVO110. (A) Schematic representation of the intergenic regions amplified by RT-PCR; numbers in parentheses indicate the sizes of the expected amplification products (numbered 1 to 6). (B) Gel electrophoresis (1.0% agarose) of RT-PCR amplicons obtained using cDNA synthesized from RNA samples. M, molecular weight DNA marker (DNA ladder, Life Technologies). Numbers indicate amplification products shown in panel A. The primer pairs used are detailed in Table S1 in the supplemental material.

The increased fitness of the GAM3 mutant during the colonization of R. necatrix hyphae (Fig. 4) and avocado roots (Fig. 5), together with the localization of the gene interrupted in this strain (GGDEF/EAL) in the proximity of a gene cluster (*cheR*, *cheW*, *cheA*, and *cheB*) possibly involved in flagellar motility/chemotaxis (Fig. 2), prompted us to investigate whether all these genes are coexpressed as an operon that could be involved in the determination of these phenotypes. Cotranscription was analyzed by real-time PCR (RT-PCR) assays performed using RNA samples isolated from LB-grown P. pseudoalcaligenes AVO110 cells. Amplification of the intergenic regions located between the sequential ORFs, with the exception of the regions upstream *cheR* and downstream the GGDEF/EAL domain-encoding gene (Fig. 2), revealed that all these genes were cotranscribed (Fig. 6).

The chemotactic responses of wild-type P. pseudoalcaligenes AVO110 and the GAM3 mutant toward R. necatrix exudates were tested in minimal medium plates (0.2% agar). However, no chemotactic movement was observed for any of these strains under the conditions tested (data not shown). We also tested the swimming ability of the GAM3 mutant in comparison to that of the wild-type strain using 0.3% King’s medium B (KB) agar plates. No significant differences in the diameters of the swimming halos (measured after 24 h, 48 h, and 72 h) were found between the strains (data not shown), indicating that the motility of the GAM3 mutant was not affected under the conditions tested.

## DISCUSSION

STM has been successfully applied to identify virulence genes in a vast number of human (40–42), animal (43, 44), and plant bacterial pathogens (45–48). In addition, this strategy has been used for the identification of genes required during the interaction of beneficial bacteria with plants (49). Here, we report the application of STM to the interaction established between a biocontrol bacteria and a fungal phytopathogen. Although complete coverage of the P. pseudoalcaligenes AVO110 genome was not achieved in this study, STM libraries composed of a lower number of mutants have been successfully applied for the identification of virulence factors in bacterial plant pathogens (47, 50) and animal pathogens (51).

### Metabolism-related genes required for growth of AVO110 in R. necatrix exudates.

Nine of the twenty-six (approximately 35%) GAM strains analyzed had interruptions in genes related to the metabolism of diverse compounds (Table 2). Strains GAM9 (*gltB*), GAM12, and GAM21 (both interrupted in *leuC*) contained disruptions of genes related to the biosynthetic pathways of the amino acids glutamate and leucine and were considered auxotrophs. These results, which are in agreement with the selection of auxotrophic strains in other STM studies (44, 46, 47), suggest that these two amino acids are limiting for the growth of P. pseudoalcaligenes in *Rosellinia* exudate-containing medium. However, we cannot rule out that mutants with disruptions in the biosynthetic pathways of other amino acids also displayed limited growth in this medium, as they were either not represented in our library or were discarded. In relation to bacterial responses to the fungal exudate, the chemotactic responses of P. putida and Pseudomonas tolaasii to Agaricus bisporus mycelial exudate have been shown to be mainly dependent on amino acids, including leucine and glutamate (13).

The *purB* gene (GAM18), which encodes an adenylosuccinate lyase involved in the biosynthesis of purines (52), has been described as essential for rhizosphere colonization by Pantoea agglomerans (53) and for infection thread formation and nodule development in Lotus japonicus induced by Mesorhizobium loti (52). In relation to the relevance of the purine biosynthetic pathways in bacterial-fungal interactions, and in agreement with our results, the P. putida *purM* gene was shown to be involved in the colonization of fungal mycelia by IVET (23). Future identification of compounds secreted into the medium by R. necatrix should shed light on the specific nutrients required for the association established between P. pseudoalcaligenes and this fungal pathogen.

### Transporters and transcriptional regulators involved in growth and survival of AVO110 in R. necatrix exudates.

The *kefA* gene, which is interrupted by the transposon in GAM14, encodes a potassium-efflux system involved in bacterial protection against the detrimental effects of electrophilic compounds via acidification of the cytoplasm (54). On the other hand, the interrupted gene in strain GAM6 (*dppA*) encodes a periplasmic dipeptide-binding protein required for dipeptide transport and chemotaxis (55). Despite the relevance of these genes in bacterial physiology, their role in bacterial interactions with fungi remains to be elucidated.

Several transcriptional regulators were found to be required for the growth and persistence of P. pseudoalcaligenes AVO110 in fungal exudate (Table 2). Other transcriptional regulators have been related to bacterial adaptation and tolerance to adverse conditions generated in the proximities of fungal hyphae (14). For example, the *copRS* operon (GAM20) and the *ntrB* gene (GAM8) are involved in other bacteria with metal resistance and responses to changes in nitrogen balance, respectively (56, 57). The possible role of the remaining transcriptional regulators identified in this study (*algQ* and *colS*) in the ability of AVO110 to survive in fungal exudates is discussed below.

### AVO110 genes involved in the colonization of R. necatrix hyphae.

The gene interrupted by the transposon in strain GAM2 (DLP-encoding gene) had reduced transcript levels after wild-type P. pseudoalcaligenes AVO110 was exposed to fungal exudates (Fig. 3). On the other hand, the inactivation of this gene in GAM2 resulted in a reduced ability of this mutant to grow and survive in fungal exudates (Fig. 1) and to persist on R. necatrix hyphae (Fig. 4), indicating that this DLP is involved in the ability of AVO110 to establish a close association with R. necatrix.

The gene interrupted by the transposon in GAM3, which encodes a GGDEF/EAL domain-containing protein, is possibly involved in the metabolism of cyclic di-GMP (c-di-GMP). This second messenger has been reported to regulate a wide range of functions, including the switch between the planktonic and sessile lifestyles, bacterial adhesion and motility, responses to root exudate, colonization of host tissues, and virulence (58–61). Moreover, we have shown that this gene is coexpressed with a *cheRWAB* cluster (Fig. 6), possibly involved in flagellum-mediated chemotactic responses and motility (62). However, no differences in swimming motility were found between the GAM3 mutant and the wild-type strain. Inactivation of Rup4959, a P. putida GGDEF/EAL domain-containing protein induced by root exudate, altered neither the swimming ability of the strain nor its ability to interact with plants (35). On the other hand, and although the expression of the *cheRWAB* genes is probably not affected by the transposon insertion in the GAM3 mutant (Fig. 2), inactivation of *che*-related genes generally results in the alteration of flagellum-mediated motility toward chemoattractants (62). Although we tested the chemotactic movement of wild-type P. pseudoalcaligenes AVO110 and the GAM3 mutant toward R. necatrix exudates, this response was not observed for either of the two strains under the conditions tested (data not shown). The identification of rhizosphere and fungal exudate attractants involved in swimming chemotaxis and c-di-GMP signaling is needed to gain greater insight into the role of these mechanisms in the biological control of fungal pathogens.

The gene interrupted by the transposon in strain GAM24 (*colS*) had increased transcript levels after P. pseudoalcaligenes AVO110 was exposed to fungal exudates (Fig. 3). In addition, this mutant showed a reduced ability to colonize both R. necatrix hyphae (Fig. 4) and the avocado rhizosphere (Fig. 5). The *colS* gene encodes a sensor element of the two-component system ColR/ColS, which is involved in bacterial outer membrane permeability (31, 63). In agreement with the reduced ability of strain GAM24 to colonize the avocado rhizosphere, a P. fluorescens *colS* mutant has been shown to be defective in competitive root colonization (31).

### AVO110 genes required for efficient colonization of avocado roots but not for establishment in R. necatrix hyphae.

Although strains GAM22 (*recB* mutant) and GAM26 (*algQ* mutant) were outcompeted by the parental strain in BM-RE medium (Fig. 1), both mutants were as competitive as the wild-type strain during the colonization of R. necatrix hyphae (Fig. 4), suggesting that these two genes are not essential during the physical interaction of P. pseudoalcaligenes AVO110 with fungal hyphae. However, their ability to colonize the avocado rhizosphere was reduced in comparison to that of the parental strain (Fig. 5). The *recB* gene encodes the helicase protein forming the RecBCD holoenzyme, which is involved in homologous recombination and in repair of bacterial DNA damage. This enzyme was found to be related to efficient biofilm formation and host colonization (64, 65). On the other hand, AlgQ regulates the production of alginate, a polysaccharide known to be involved in biofilm protection and colonization of plant tissues (28, 66). In addition, AlgQ has been described as a global regulator in P. aeruginosa, upregulating siderophore synthesis and downregulating quorum sensing regulation (29).

In summary, this application of STM allowed us to identify genes of the bacterial biocontrol agent P. pseudoalcaligenes AVO110 that are required for growth and survival in the presence of fungal exudates, some of which are also essential for the colonization of fungal hyphae and/or plant root surfaces. Several metabolic pathways were highlighted as essential for the interaction of this bacterium with R. necatrix, such as those related to the metabolism of hydrocarbons, including acyclic terpenes, amino acids, fatty acids, and aromatic compounds. In addition, the relevance of a dipeptide transporter, metal resistance, protection against acidification of the cytoplasm, and maintenance of nitrogen balance were also noted in this study as essential for bacterial survival under the influence of fungal exudate. The bacterial genes identified here as required for the colonization of both fungal hyphae and plant roots are likely involved in membrane dynamics or the cross talk between c-di-GMP signaling and chemotaxis. Finally, an additional set of two genes, which are perhaps related to responses to chemical stress and biofilm formation, was also identified as required for the colonization of the avocado rhizosphere. Further functional characterization of these genes and of the compounds secreted by R. necatrix into the medium may promote a better understanding of the multitrophic interactions established among bacterial biocontrol agents, the plant rhizosphere, and the mycelia of soilborne pathogens.

## MATERIALS AND METHODS

### Bacterial strains, plasmids, media, and growth conditions.

A pool of DNA sequence-tagged pUTmini-Tn*5*Km plasmids, E. coli strains CC118(λ*pir*), S17-1 λ*pir*, and DH5α, and protocols for STM were kindly provided by D.W. Holden (Imperial College, London). The original bacterial strains and plasmids used in this study are listed in Table 1. P. pseudoalcaligenes strains were grown at 28°C in King’s medium B (KB) (67), lysogeny broth (LB) medium (68), standard succinate medium (SSM) (69), or super optimal broth (SOB) medium (70). E. coli strains were grown at 37°C in LB or SOB medium. Solid and liquid media were supplemented, when required, with the following antibiotics (μg · ml^−1^) for *Pseudomonas*/E. coli strains: ampicillin (Amp) 100; Km, 25; nitrofurantoin (Nf), 50; Gm, 25; and cycloheximide (Ch), 50. The P. pseudoalcaligenes derivatives selected in this study that contained a mini-Tn*5*Km2 transposon are listed in Table 2. The primers used in this study are listed in Table S1 in the supplemental material. R. necatrix Rn400 was grown at 25°C on KB agar plates and stored at 4°C in water. Fungal mycelium was routinely replicated on KB plates to test viability every 6 months. BM minimal medium (4) was used for growing R. necatrix mycelia to obtain fungal exudate-containing medium (1) as described below (see “Preparation of BM medium containing fungal exudates”).

### Preparation of BM medium containing fungal exudates.

R. necatrix Rn400 was grown at 25°C on BM agar plates until the surface was completely covered by the fungus. To obtain liquid medium containing fungal exudates, fungal mycelium was collected from one plate, inoculated in 200 ml of minimal BM medium, and incubated at 25°C for 2 weeks without shaking. Fungal mycelium was removed from the culture medium via filtration using sterile filter paper (RM 2354252; Albet) in reams of 73 g · m^−2^ (1). The final pH of the fungal exudate-containing medium (BM-RE) was 7.11.

### Generation of a unique-tag marked library of Pseudomonas pseudoalcaligenes mutants.

A library of signature-tagged transposon mutants of P. pseudoalcaligenes was constructed as described in reference 71 with minor modifications. The pool of tagged pUTmini-Tn*5*Km2 vectors was transferred from E. coli S17-1 λpir to P. pseudoalcaligenes by plate conjugation mating as previously described (1). The transposition frequency of mini-Tn*5*Km2, which confers resistance to Km, was 6.8 × 10^−6^ transconjugants/receptor in the genome of P. pseudoalcaligenes AVO110 using biparental mating delivery. The constructed random transposition library consisted of 38 different 96-well microtiter trays, containing a total of 3,408 P. pseudoalcaligenes mutants. Individual colonies were challenged on LB-Amp plates to discard P. pseudoalcaligenes transconjugants harboring the plasmid vector.

Agarose gel electrophoresis and other standard recombinant DNA techniques were performed as described previously (72). Genomic DNA was extracted using the JetFlex extraction kit (GenoMed, Löhne, Germany) according to the manufacturer's instructions. Single and random transposon insertions into the genomes of the mutant strains were confirmed by Southern hybridization (73).

### Colony blots.

To fix total DNA from the colonies, overnight cultures of P. pseudoalcaligenes mutant strains grown on LB-Km microtiter plates were transferred onto nylon membranes placed on LB-Km agar plates using a 48-pin replicator (Sigma-Aldrich, St. Louis, MO, USA). Colony blots were performed as previously described in reference 17.

### STM screening.

STM screening was carried out by testing 76 pools of ≤45 mutants mixed with a negative control (wild-type P. pseudoalcaligenes AVO110) and a positive control (a mini-Tn*5*Km2-tagged AVO110 derivative selected by its ability to grow and survive on both LB and BM-RE media). The input pools were generated by mixing 100 μl of cultures grown for 24 h at 28°C on microtiter plates containing LB (two wells per pool incubated with the negative control) and LB-Km (AVO110 mutants and one well per pool incubated with the positive control). Next, the mixtures were washed twice with NaCl 0.9% and adjusted to an OD_600_ of 0.1 (approximately 10^6^ CFU · ml^−1^). Afterwards, 100 μl of these suspensions was used to inoculate 5 ml of BM medium amended with 5 ml of BM-RE medium (10^3^ to 10^4^ CFU · ml^−1^), which was incubated at 28°C and 225 rpm. After 2 days, mutant cells were recovered from the medium (10^6^ to 10^7^ CFU · ml^−1^) to generate the output pool (17). Finally, the 40-bp probes were purified using Microspin G-50 columns (GE Healthcare, Buckinghamshire, UK). DNA hybridizations on colony blots to ^32^P-labeled probes were carried out as described in reference 17. A schematic representation of the STM selection process is shown in Fig. S1.

### Determination of transposon insertion sites.

Genomic DNA from selected mutants was digested with EcoRI and ligated into pBluescript II SK digested with the same restriction enzyme. Ligation reactions were used to transform DH5α by heat shock (70), and single Km-resistant colonies were selected. Plasmids showing DNA fragments of at least 1.8 kb were purified using a NucleoSpin Plasmid Quick Pure kit, and the DNA regions flanking the transposons were sequenced using primer P7 (Table S1) (71). Automated DNA sequencing was performed by SecuGen (Madrid, Spain). The raw sequences were analyzed by general BLASTn searches against NCBI-deposited sequences.

The DNA context surrounding each of the genes interrupted by the transposons in GAM strains was analyzed using the Artemis 13.2.0 genome browser/editor (74) and BLAST searches (http://blast.ncbi.nlm.nih.gov/Blast.cgi).

### Draft genome sequencing and annotation.

Genomic DNA from P. pseudoalcaligenes AVO110 was extracted from bacterial cells grown overnight in LB medium supplemented with Nf at 25°C. DNA was extracted using a genomic DNA purification JetFlex kit (GenoMed GmbH, Löhne, Germany) according to the manufacturer’s instructions. The DNA sample was further purified by first extracting with phenol-chloroform-isoamyl alcohol (25:24:1) and then extracting with chloroform-isoamyl alcohol (24:1). DNA was precipitated with one-tenth volume of 3 M sodium acetate and two volumes of 100% ethanol and resuspended in Milli-Q water. NanoDrop measurements gave a concentration of 315 ng · μl^−1^ (315 μg of DNA in total) with an *A*_260_/*A*_280_ of 1.79. The draft genome of P. pseudoalcaligenes AVO110 was sequenced using the Illumina HiSeq 2000 platform at BGI Tech Solutions Co., Ltd. (Hong Kong), and paired-end reads with insert sizes of 500 bp were assembled using SOAP *de novo* software (75, 76). Statistics regarding the assembly results are summarized in Table S2. Automatic annotation of the draft genome was obtained using the RAST server (77).

### *In vitro* competition assays.

*In vitro* competition assays were performed as previously described (78). Competitive indexes (CIs) were calculated by dividing the output ratio (CFU mutant/CFU wild type) by the input ratio (CFU mutant/CFU wild type). The LB and BM-RE competition indexes reported (CI_LB_ and CI_BM-RE_, respectively) are the means from three independent experiments ± standard deviation. Data were analyzed as described below (see “Statistical analysis”).

### Mini-Tn*7* tagging of GAM strains.

The *gfp* delivery plasmid pBK-mini-Tn*7*-Gm-*gfp* and the helper plasmid pUX-BF-13 were introduced into GAM strains by tetraparental mating as described in reference 79. Cells of the corresponding recipient strain, E. coli donor strain XL1-Blue/pBK-mini-Tn*7*(Gm)_PA1/04/03_-*gfp*, and helper strains E. coli SM10(pUX-BF13) and E. coli HB101(pRK600) (Table 1) were mixed at a 3:1:1:1 ratio. PCR analyses on chromosomal DNA of the corresponding *gfp*-tagged strains (Table 1) were performed to determine whether the mini-Tn*7* insertions in GAM strains (Table 2) occurred at a specific site without gene disruption. In agreement with data reported for other *Pseudomonas* strains, PCR products that were approximately 150 bp long were amplified from derivative GAM strains using primers Tn*7-glmS* and Tn*7*-R109 (80, 81).

### RT-PCR and quantitative RT-PCR assays.

For RT-PCR, P. pseudoalcaligenes AVO110 cells were grown in LB to an optical density at 600 nm (OD_600_) of 0.5. The cells were pelleted and stored at −80°C. Total RNA was extracted using the TriPure isolation reagent (Roche Applied Science), as described previously (82). RNA concentration was determined spectrophotometrically, and its integrity was assessed by agarose gel electrophoresis. Total RNA was treated with a Turbo DNA-free kit (Applied Biosystems, Foster City, CA, USA), as detailed by the manufacturer’s instructions. DNA-free RNA was reverse transcribed using random hexamers included in the iScript cDNA synthesis kit (Bio-Rad, Hercules, CA, USA). cDNA was used as a template to amplify intergenic regions by PCR, using GoTaq polymerase (Promega) and the primers indicated in Table S1. PCR products were analyzed by 1% agarose gel electrophoresis.

Quantitative real-time PCR (qRT-PCR) assays of P. pseudoalcaligenes AVO110 genes were performed using iQ SYBR green supermix (Bio-Rad, CA, USA) as follows. P. pseudoalcaligenes AVO110 was grown overnight in LB. The next day, the cells were diluted in 100 ml of LB medium to an OD_600_ of 0.05 and grown to an OD_600_ of 0.5. Cells were washed with NaCl 0.9% three times and inoculated in 100 ml of BM-RE medium to start the induction. Three samples of 25 ml each were extracted from this volume, and RNA extraction was carried out at time zero and after 4 h and 24 h of incubation in BM-RE medium. RNA extraction and cDNA synthesis were performed as described above. Target cDNAs from the experimental samples were amplified in triplicates in separate PCRs using 0.3 M each primer, GAM2QFwd/GAM2Qrev for GAM2, GAM3QFwd/GAMQrev for GAM3, GAM22QFwd/GAM22Qrev for GAM22, GAM24QFwd/GAM24Qrev for GAM24, and GAM26QFwd/GAM26Qrev for GAM26 (Table S1). The PCR amplicons were between 100 bp and 200 bp in length. Transcriptional data were normalized to the housekeeping gene *rpo*D from P. pseudoalcaligenes AVO110. After the normalization, expression fold changes at 4 h and 24 h were calculated with respect to the gene expression obtained right after the transfer to BM-RE medium (time zero). qRT-PCR values were the means from three biological replicates with three technical replicates ± standard deviations. Data were analyzed as described below (see “Statistical analysis”).

### Bacterial colonization on fungal mycelia.

Colonization of fungal mycelia by GAM strains was determined in competition with the wild-type strain P. pseudoalcaligenes AVO110 as previously described by (22) with slight modifications (1). Agar discs obtained from potato-dextrose agar (PDA) plates containing actively growing R. necatrix mycelia were placed onto BM agar plates, on which AVO110 (1) and GAM strains are not able to grow, covered with a cellophane layer, and incubated for 10 days at 25°C. After this period, 1.5 ml of bacterial suspension previously washed twice with NaCl 0.9% and containing 10^4^ to 10^5^ CFU · ml^−1^ was equally distributed along the surface of each plate, and the plates were left to dry under a flow chamber for 2 h. After 6 days of incubation at 25°C, the cellophane layers containing actively growing fungal and bacterial strains were placed in sterile plastic bags, weighed, transferred to a lab blender, and homogenized for 2 min with 2 ml of sterile NaCl 0.9% to release the bacteria. Suspensions were serially diluted and plated on KB supplemented with Nf, Ch, and Km (GAM strains) and Ch and Nf for total bacterial counts. Bacterial counts were obtained after 48 to 72 h at 25°C. P. pseudoalcaligenes wild-type strain counts were calculated as a ratio of bacterial total counts to GAM counts.

CIs were calculated as described above. The CIs reported are the means from three technical replicates from three independent experiments ± standard errors. Data were analyzed as described below (see “Statistical analysis”).

### Bacterial colonization on avocado roots.

P. pseudoalcaligenes AVO110-Km, GAM2-Gm, GAM3-Gm, GAM22-Gm, GAM24-Gm, and GAM26-Gm derivative strains were used. Six-month-old avocado seedlings of the commercial rootstock cv. Walter Hole (Brokaw nursery, Spain) were disinfected and inoculated with bacteria as previously described in reference 12 using suspensions containing 10^3^ to 10^4^ CFU · ml^−1^. Plants were placed in nonsterile vermiculite and grown in a growth chamber at 24°C, 70% relative humidity, and 16 h of daylight. Bacterial recovery from the roots was performed as follows. Three seedlings were removed from the vermiculite and processed independently at 7, 15, 30, 48, and 72 days of plant growth. Roots were separated from the plant, placed into sterile plastic bags, weighed, and subsequently transferred to a lab stomacher and homogenized for 4 min with 2 ml of sterile phosphate-buffered saline (PBS; 0.1 M, pH 7.2) per gram of fresh root material. Suspensions were serially diluted and plated on LB supplemented with Nf and Gm for counts of GAM strains and Nf and Km for counts of the wild-type strain. Bacterial counts with appropriate colony morphology and antibiotic resistance were obtained after 48 to 72 h at 25°C. Data represent the averages from at least three independent plants per sampling point ± standard errors.

### Statistical analysis.

Data were analyzed using SPSS software v.22 (SPSS Inc., Chicago, IL, USA). Competition index values resulting from *in vitro* competition assays and qRT-PCR values were analyzed using a Student’s *t* test and the following null hypothesis: mean index was not significantly different from 1.0 (using *P* values of 0.05). Competition index values obtained from bacterial colonization on fungal mycelia were subjected to a one-way analysis of variance (ANOVA) followed by Tukey’s honestly significant difference (HSD) test with the correction of Bonferroni (*P* = 0.05).

### Accession number(s).

The sequences surrounding the transposon in a selection of GAM strains were deposited in National Center for Biotechnology Information (NCBI) (https://www.ncbi.nlm.nih.gov/) under the following accession numbers: GAM2 (KX863700), GAM3 (KX858709), GAM22 (KX906975), GAM24 (KX858711), and GAM26 (KX858710).

## Supplementary Material

Supplemental file 1
