# Supplementary material for: Response of the Biocontrol Agent Pseudomonas pseudoalcaligenes AVO110 to Rosellinia necatrix Exudate
Source: Appl Environ Microbiol. 2019 Jan 23;85(3):e01741-18. doi: 10.1128/AEM.01741-18 (PMC6344628; doi:10.1128/AEM.01741-18)
Supplement: Supplemental file 1 [file be9a8927b1a47c7b8652ed84a3bed342_AEM.01741-18-s0001.pdf]

Supporting Information Fig. S1

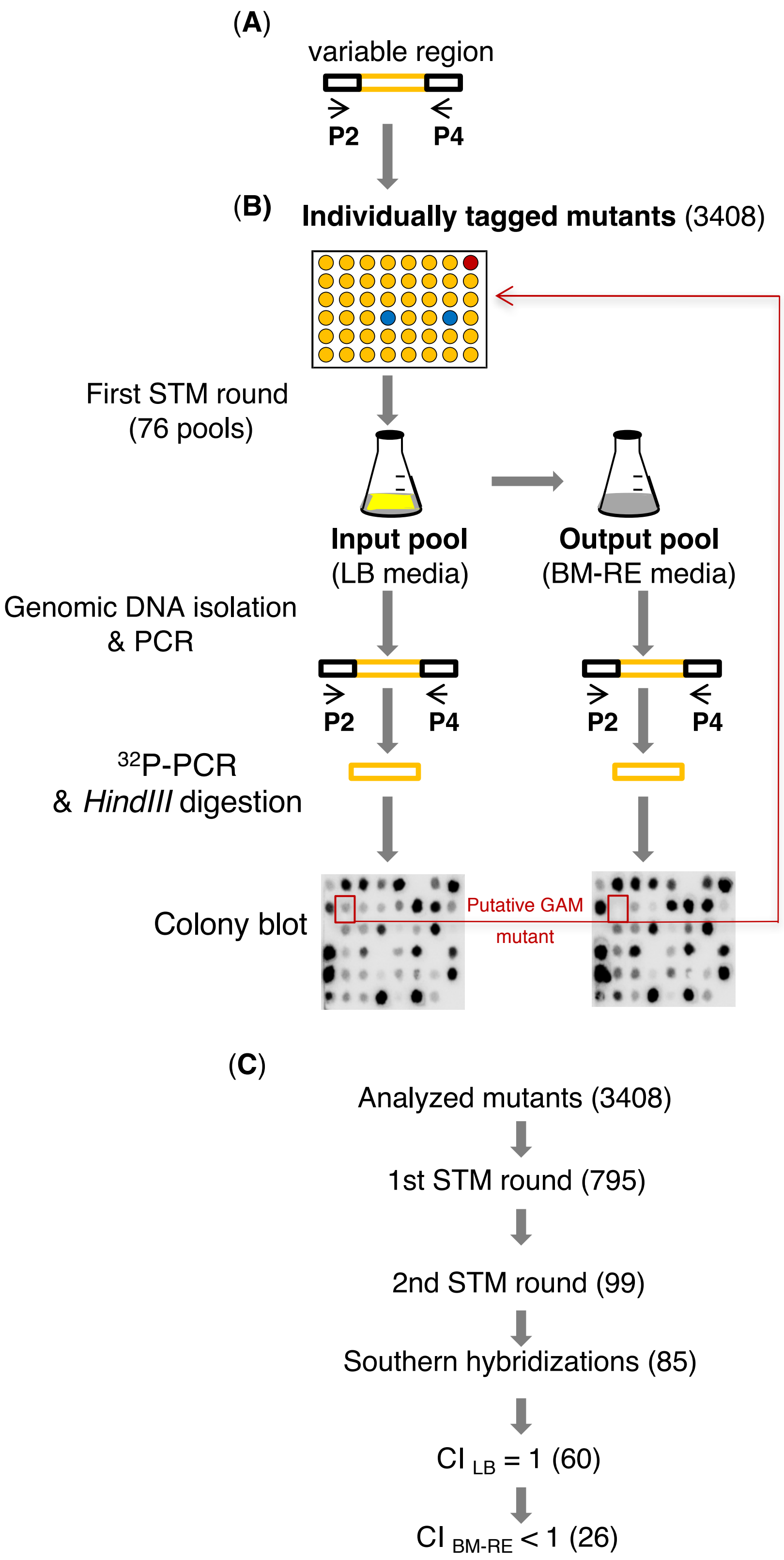

Figure S1: Schematic representation of the STM screening strategy followed in this study for the selection of *P. pseudoalcaligenes* AVO110 mutants with reduced competitiveness in minimal BM medium supplemented with *R. necatrix* exudates (BM-RE medium). (A) DNA sequence tags in miniTn*5*Km2 transposons. Each tag contains a particular 40 bp variable central region and two 20 bp invariant flanking arms, which bind P2 and P4 primers. (B) Each 98 well Petri dish contained a pool of 48 bacterial strains, composed by 45 *P. pseudoalcaligenes* AVO110 miniTn*5*Km2-tagged strains, two negative controls (wild-type *P. pseudoalcaligenes* AVO110, blue filled circles) and one positive control (a miniTn5Km2-tagged AVO110 derivative selected by its ability to growth and survive on both LB and BM-RE, red filled circle). Genomic DNA was isolated from either bacterial mixtures grown in LB (input pool) or bacterial mixtures recovered from BM-RE after 48 hours of incubation at 28 ºC (output pool). Probes were amplified by PCR using primers P2 and P4, and the two arms were released by digestion with HindIII. Two membranes with colonies, duplicated from the stored plate, were hybridized with the generated input and output probes. Mutants showing reduced signal with the output probe compared with the input probe (red boxes) had potentially altered ability to grow and/or survive in BM-RE media. Selected mutants were used within new pools, mixed with other random mutants, for a second STM round (for details, see Materials and Methods). (C) Workflow and number of mutants selected at each step.

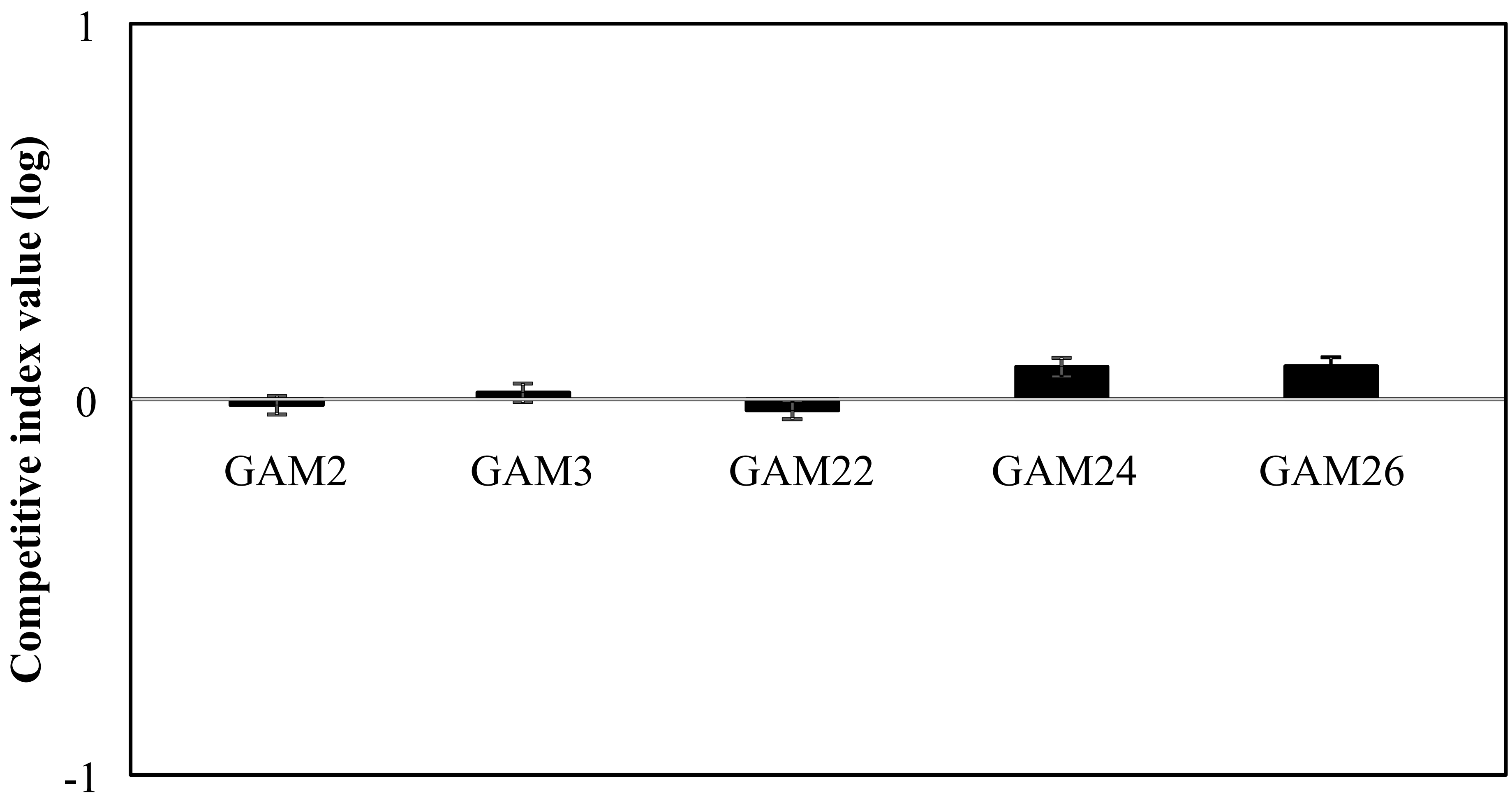

Fig. S2. Competition assays of *Pseudomonas pseudoalcaligenes* growth-attenuated mutants (GAM) tagged with a mini-Tn*7*-Gm-Gfp transposon (GAM-Gm strains, **Table 1**). Competitive index values (CIs) are shown for mixed inoculations in lysogenic broth (LB) medium of *P. pseudolacaligenes* AVO110 tagged with a mini-Tn*7*-Km transposon (AVO110-Km, **Table 1**), encoding kanamycin resistance, and the GAM-Gm AVO110 derivatives, which also carry a miniTn*5*-Km transposon. The CIs shown, which are in all cases no significantly different from unity, are the mean of three technical replicates from three independent experiments. The error bars represent the standard deviation from the average. Statistical analyses were performed using Student's t-test ($p = 0.05$).

Table S1. Primers used in this study

**Oligonucleotide[a]**	**Sequence (5' to 3')**
GAM2QFwd	CCACCAATCCGATCAAAGAG
GAM2QRev	GAACCGACTGAGTACGAAAACC
GAM3QFwd	AGCGCGATAGCTGGTCTATG
GAM3Qrev	TTGAGGATGGTCTTGTGCTC
GAM22QFwd	CATCAACGCCGAACTGTG
GAM22Qrev	GCCACAGGAAAAGTGAAACC
GAM24QFwd	GGTTGACCTCGAAGCGACAG
GAM24Qrev	GTGTAGGCATTCCCGAAGAG
GAM26QFwd	GTTCGAGGTGGAGTTGCTG
GAM26Qrev	ACTGGAAGACTGCCTGATCG
rpoDQFwd	GCATCCTCGGTGAATACCAG
rpoDQRev	CCATCTCCTTCTTCTTCGTC
P7	CAAGCAGAAGACGGCATACGAGAT
P2	CATATGAATATCCTCCTTAG
Tn7-*glmS*	CTCAAGTCGAACCTGCAGGAAGTC
Tn7rev109	CAGCATAACTGGACTGATTTCAG
RTGAM3.1Fwd	ACTCTACCTGGAGCCCGATT
RTGAM3.1rev	CGATCCGTCGCTCTAGCTC
RTGAM3.2Fwd	ACCAATGGACAGTGCTGGAC
RTGAM3.2rev	ACCTCACGGAACAACGACTC
RTGAM3.3Fwd	GCCAATGCCTATCTGGTCAA
RTGAM3.3rev	AGGAAGTCCTGCAACACCTG
RTGAM3.4Fwd	CATCGCGACCTATCTCAACA
RTGAM3.4rev	AGAGCTGGTAGCCGTCCAT
RTGAM3.5Fwd	CGTGGTATACCGCTTCATCA
RTGAM3.5rev	GTTTCGCAGTTCAGGCACTT
RTGAM3.6Fwd	AGCCGCTAAAAAGTTGTCCA
RTGAM3.6rev	GTGCACCTTGTTCACCAGAA

[a]Primers used for RT-PCR analysis of the *cheRWAB*-GGDEF/EAL operon in *P. pseudoalcaligenes* AVO110 are named RTGAM3 followed by a number, indicating the intergenic region amplified by RT-PCR (**Fig. 6**).

Table S2. General characteristics of the draft genome assembly of *Pseudomonas pseudoalcaligenes* AVO110

	Scaffolds	*Contigs*
Total Number	16	70
Total length (bp)	4.945.220	4.940.426
Maximum length (bp)	1.448.505	448.229
Minimum length (bp)	515	240
G+C content (%)	65	65
